# Arterial Stiffness and Cardiovascular Therapy

**DOI:** 10.1155/2014/621437

**Published:** 2014-08-07

**Authors:** Miodrag Janić, Mojca Lunder, Mišo Šabovič

**Affiliations:** Department of Vascular Diseases, University of Ljubljana Medical Centre, Zaloška 7, SI 1000 Ljubljana, Slovenia

## Abstract

The world population is aging and the number of old people is continuously increasing. Arterial structure and function change with age, progressively leading to arterial stiffening. Arterial stiffness is best characterized by measurement of pulse wave velocity (PWV), which is its surrogate marker. It has been shown that PWV could improve cardiovascular event prediction in models that included standard risk factors. Consequently, it might therefore enable better identification of populations at high-risk of cardiovascular morbidity and mortality. The present review is focused on a survey of different pharmacological therapeutic options for decreasing arterial stiffness. The influence of several groups of drugs is described: antihypertensive drugs (angiotensin-converting enzyme inhibitors, angiotensin receptor blockers, calcium channel blockers, beta-blockers, diuretics, and nitrates), statins, peroral antidiabetics, advanced glycation end-products (AGE) cross-link breakers, anti-inflammatory drugs, endothelin-A receptor antagonists, and vasopeptidase inhibitors. All of these have shown some effect in decreasing arterial stiffness. Nevertheless, further studies are needed which should address the influence of arterial stiffness diminishment on major adverse cardiovascular and cerebrovascular events (MACCE).

## 1. Introduction

The world population is aging so the number of old people is continuously increasing [1, 2]. With increasing age, arterial structure and function change, progressively leading, among other deteriorations, to arterial stiffening [3, 4]. One of the most important parameters most commonly measured and understood, being also the best surrogate for arterial stiffness, is pulse wave velocity (PWV) [5–7]. In a recent meta-analysis, aortic PWV was found to improve cardiovascular event prediction in models that included standard risk factors (arterial hypertension, smoking, diabetes, etc.) and might therefore enable better identification of high-risk populations [8, 9]. Even though this data exists, there is still no pharmacological approach regularly used in clinical practice aiming to decrease arterial stiffness. In other words, the therapeutic approach does not aim at arterial stiffness decrease* per se.* Although evidence of the importance of PWV is growing, there was no study reported in which a decrease of cardiovascular mortality due to reducing arterial stiffness by pharmacologic approaches had been observed. Nevertheless, we believe that there is sufficient proof of PWV being an important cardiovascular risk factor and that such a study is very much needed. Therefore, in what follows we review all known pharmacological approaches capable of decreasing arterial stiffness. Importantly, it should be noted that the effects of pharmacologic agents on stiffness are usually slight or modest, but not substantial. Thus, new therapeutic approaches to decrease arterial stiffness are highly desirable.

## 2. Pathophysiological Aspects of Arterial Stiffness

Conductive arteries propel the pressure wave generated by the heart, that is, the ejection of blood from the left ventricle. This wave is reflected at the impedance mismatch points (junctions of large conduit arteries, high-resistance arteries, and bifurcations), from where it travels backwards to the heart. Consequently, the observed generated wave is the sum of the forward travelling wave (moving from the heart) and the reflected wave (travelling backwards towards the heart) [10]. In young healthy subjects who have compliant arteries the reflected waves return to the ascending aorta at the time of diastole, thus leading to pressure amplification in this part of cardiac cycle, leading to an increase in diastolic blood pressure (DBP) [11]. As pulse waves travel faster in stiffer arteries, PWV measurement is consequently the best surrogate for arterial stiffness evaluation in everyday practice. It also increases with age and is a predictor of cardiovascular risk. It has been calculated that an increase in PWV by 1.0 m/s increases the risk of cardiovascular events by 14% [12].

The low blood pressure elastic modulus of the elastin component of arterial media dominates the mechanical behavior of the arterial wall, making it distensible [10]. At higher blood pressures, the wall is less extensible, due to the low elastic modulus of the collagen component of the arterial media that dominates at these pressures [13]. It can be concluded that at low blood pressures a small amount of collagen fibers is recruited. When the blood pressure rises, more and more collagen fibers are engaged, the elastin component having relatively less influence, leading to sufficient support of the arterial wall and stabilization of aortic root distension. To sum up, arterial wall compliance and distensibility progressively decrease with increasing blood pressure. Blood pressure-dependent changes in elastic modulus are nonlinear; that is, the change in elastic properties is much greater at high blood pressures than at low blood pressures [10]. Blood pressure increase leads to PWV increase. The consequence is pressure wave propagation, which is a result of the increase in amplitude of the wave travelling from the heart. It means that the top of the wave travels faster than the rest of the wave. This leads to the physiological findings of a consistent difference between blood pressure values in the ascending aorta and brachial artery. In young healthy subjects, the difference between pulse pressure (PP) and systolic blood pressure (SBP) in the ascending aorta and at the level of brachial artery can be as much as 20 mmHg, while in patients treated for hypertension it is considerably lower (6 to 11 mmHg) [11].

The arteries become stiffer with increasing age and disease (e.g., hypertension, chronic kidney disease, diabetes, and atherosclerosis). Increased stiffness results from structural changes, such as fragmentation of elastin, an increased amount of collagen, arterial calcification, glycation of both elastin and collagen, and cross-linking of collagen by advanced glycation end- products (AGE) [14–17]. These changes could be measured quantitatively in the pathology department. On the other hand, clinical evaluation of arterial mechanical properties is far more complex and a complete description of the strain-stress relationship* in vivo* is not possible due to uncertainties arising from nonlinear behavior, viscoelasticity, anisotropy, active tone, residual stresses, and tethering [18]. The methods most commonly used measure transit times between different sites in the arterial tree and calculate arterial PWV and measure local arterial compliance, the distensibility or stiffness index, and also the augmentation index (Aix) [19].

**Figure 1:**
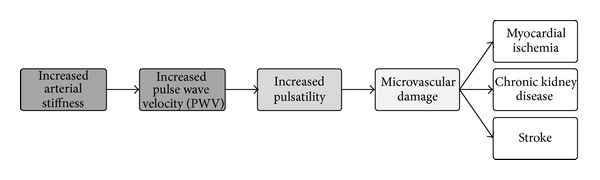
Flowchart describing the effect of increased arterial stiffness on end organ damage.

Increased arterial stiffness leads to increased PWV and central arterial pressure, resulting in higher arterial pulsatility. The latter leads to damage of the microcirculation in several organs, especially the highly perfused ones, such as myocardium, the kidneys, and brain (Figure 1). Taking into account that the population is aging, stiffness is an important factor in pathophysiological aspects [20].

## 3. Endothelial Function and Arterial Stiffness

**Figure 2:**
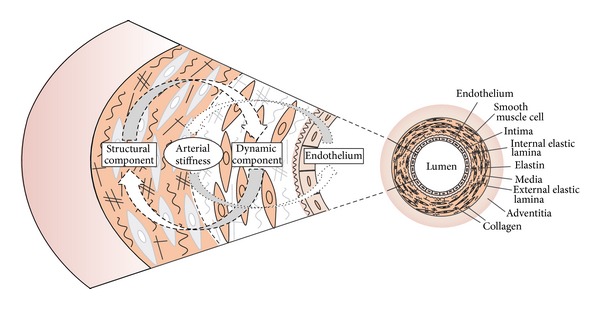
Vicious cycle—the connection between the dynamic and structural components of arterial stiffness.

Endothelial function and arterial stiffness are two different aspects of arterial disease, which are interconnected as their pathophysiological background is similar. Nitric oxide (NO) has been shown to contribute importantly to arterial compliance or distensibility [21]. Arterial stiffness can be regarded as composed of two distinct components: a structural and a dynamic component. These two are obviously interconnected. The structural component is represented by the collagen and elastin fibers in arterial media, as well as other connecting molecules [22]. The dynamic component is represented by the tone of smooth muscle cells, also in the arterial media. This tone is dependent on vasoactive substances released from the endothelium [23]. As mentioned above, the artery becomes stiffer due to an increase in the collagen-elastin ratio. On the other hand, the stiffer the artery gets, the greater the hemodynamic load to which its endothelium is exposed, resulting in its earlier damage. The dynamic and structural components of arterial stiffness are interconnected and lead to a vicious cycle (Figure 2).

## 4. Influence of Drugs on Arterial Stiffness

According to the literature, several drugs have been shown to influence arterial stiffness: antihypertensives, statins, peroral antidiabetics, AGE cross-link breakers, anti-inflammatory drugs, endothelin-A receptor antagonists, and vasopeptidase inhibitors. The majority of them act predominantly on the dynamic component of arterial stiffness and to a lesser extent on the structural component in arterial wall remodeling, whereas only AGE cross-link breakers act directly on the structural component. Influence of particular drugs or drug groups on arterial stiffness is summarized in Table 1.

### 4.1. Antihypertensive Drugs

Antihypertensive drugs have been implicated in arterial stiffness diminishment but vary in their degree of effect. The various antihypertensive drug classes have been more or less extensively evaluated in this regard. Unequivocally, the renin-angiotensin system inhibitors proved to be superior to all other antihypertensive drugs in reducing arterial stiffness. There are different reasons for and understanding of this phenomenon, but the most probable explanation lies in the profibrotic action of the renin-angiotensin system, as the turnover of the extracellular matrix in the arterial wall* per se* leads to a change in the properties of the vessel [24]. In what follows, antihypertensive drugs are listed according to the amount of evidence and their effect on arterial stiffness.

#### 4.1.1. Angiotensin-Converting Enzyme Inhibitors

There had been some evidence that angiotensin-converting enzyme (ACE) inhibitors lead to arterial compliance improvement [25], but it was the REASON (pREterax in regression of Arterial Stiffness in a contrOlled double-bliNd) study that first evaluated the long-term influence of these drugs on arterial stiffness. This study was performed using the perindopril/indapamide combination and compared it to the use of atenolol. The former combination proved to be more efficacious in reducing systolic blood pressure as well as pulse pressure. The effect was more pronounced in the central indices. Interestingly, PWV showed a similar reduction in both groups, while on the other hand Aix was more significantly reduced in the combination group. The effect obtained in the combination group lasted even after 9 months of treatment without additional blood pressure reduction [26, 27]. In the ADVANCE (Action in Diabetes and Vascular Disease: Preterax and Diamicron Modified Release Controlled Evaluation) trial, the same combination of perindopril/indapamide was also evaluated, and it was this study that proved the importance of arterial stiffness and its association with cardiovascular risk [28]. The effect of ACE inhibitors on pulsatile hemodynamics in patients with stable coronary artery disease was evaluated in the PEACE (Prevention of Events with Angiotensin-Converting Enzyme Inhibition) substudy. This study showed that trandolapril moderately decreased PWV, beyond expectations and without relation to blood pressure reduction. Nevertheless, no improvement in aortic compliance or decrease in Aix was observed [29]. All the studies described were long-term studies, but even some short (evaluating acute effects) to medium (less than 6 months) term studies showed a reduction of arterial stiffness when ACE inhibitors were used [30]. These effects were obtained for most drugs in this class, that is, captopril [31], perindopril [32], trandolapril [29], enalapril [33], lisinopril [34], ramipril [35], quinapril [31, 36], and fosinopril [37]. These effects were attributed to the ACE inhibitors' capability of chronically reducing remodeling of the small arteries, leading to reduction of reflection coefficients.

#### 4.1.2. Angiotensin Receptor Blockers

Intuitively, it might be expected that angiotensin receptor blockers (ARBs or sartans) would produce the same effect as ACE inhibitors. In a trial with patients with resistant hypertension who were receiving three antihypertensive drugs in maximal dosages, including ACE inhibitors, the addition of valsartan for two weeks resulted in reduction of Aix [41]. When valsartan was compared to captopril, the two drugs equally reduced PWV as well as Aix [42]. As far as Aix reduction is concerned, losartan (see the LIFE (Losartan Intervention For Endpoint reduction in hypertension) [43] and OPTIMAAL (Optimal Trial in Myocardial Infarction with Angiotensin Antagonist Losartan) [44] studies) and candesartan [45, 46] were proven to reduce it. Other ARBs, such as valsartan (the VALUE (Valsartan Antihypertensive Long-Term Use Evaluation) study) [47–49], olmesartan [50], telmisartan [51], and eprosartan [52], were proven to reduce central blood pressure more than the systolic blood pressure and increase pulse pressure, while reducing Aix and PWV. In addition, when ACE inhibitor and sartan were combined, they proved to achieve even greater effect on PWV reduction in patients with chronic kidney disease [38].

#### 4.1.3. Beta-Blockers

Beta-blockers without vasodilating properties have been shown to have a weaker effect on arterial stiffness and central pulsatile hemodynamics than vasodilating drugs of other groups. Nevertheless, they showed the same extent of reduction of arterial stiffness* per se* as the other mentioned drugs. The mechanism of action is through heart rate reduction, as this influences the viscoelastic properties of the arterial wall. Reduced heart rate also leads to increased wave reflections, a lower reduction in aortic than brachial systolic blood pressure, and reduced pulse pressure amplification. Peripheral vasoconstriction, achieved by, for example, atenolol, is an additional mechanism responsible for the negative effect on wave reflections [74–77]. The REASON study evaluated the effect of atenolol on pulsatile hemodynamics. In this particular study central PP slightly increased, while peripheral PP drastically decreased. Also PWV decreased substantially, while Aix increased to a substantial degree. These effects were attributed to the heart rate reduction [27]. Different studies are consistent, showing that atenolol negatively affects both pulse and systolic blood pressure (by increasing them) and wave reflections, increasing Aix, but, on the other hand, reducing aortic stiffness [53]. Thus the CAFÉ (Conduit Artery Functional Evaluation) study showed the superiority of amlodipine in this regard, leading to challenging the recommendation for the use of classical beta-blockers in hypertension treatment [32]. New, increasingly prescribed agents such as nebivolol and carvedilol, that also have vasodilating properties, seem to be more effective in improving central pulsatility. These effects appear to be related to their ability to donate NO, which dilates the small resistance arteries. The effects observed lead to pulse pressure amplification, but Aix reduction [39, 74–77].

#### 4.1.4. Calcium Channel Blockers

Calcium channel blockers also lower PWV and reduce wave reflections, but to a lesser degree than renin-angiotensin inhibitors. The largest amount of evidence is for the dihydropyridine calcium channel blocker amlodipine [32, 34, 37, 46, 49, 55]. This drug was evaluated in the CAFÉ study, among other trials, where it proved to reduce central blood pressure more than peripheral blood pressure; it amplified pulse pressure and reduced Aix and PWV, thus displaying its destiffening effect [32]. Similar results were obtained for the other calcium channel blockers that were evaluated, namely, azelnidipine [56], barnidipine [57], nitrendipine [58], felodipine [55], lercanidipine [59], and verapamil [60].

#### 4.1.5. Diuretics

Diuretics seem to have no beneficial effect on pulsatile hemodynamics. Many agents were studied, including hydrochlorothiazide, which showed a neutral effect on reduction of central blood pressure and a neutral effect on pulse pressure amplification [37, 55]. Consistent data is available for bendrofluazide [34, 59, 61, 113] and indapamide [37, 46, 113, 114], which also have a neutral effect on Aix and PWV, respectively.

#### 4.1.6. Aldosterone Antagonists

The aldosterone antagonist spironolactone proved to reduce PWV and Aix when adjusted for blood pressure, compared to bendrofluazide [61]. The beneficial effect of spironolactone in this regard was also obtained in early stage chronic kidney disease and in patients with nonischemic dilated cardiomyopathy [62, 63]. Similar results were obtained for eplerenone when compared to amlodipine, but its effect proved to be much greater in reducing vascular stiffness and the collagen-elastin ratio when compared to atenolol [97, 98]. These effects were not observed in chronic kidney disease patients stages 3 and 4 [99].

#### 4.1.7. Direct Renin Inhibitors

Direct renin inhibitor, that is, aliskiren, the only one available, was evaluated in diabetes mellitus type 1 and type 2 patients where it reduced PWV as well as Aix, thus showing a beneficial destiffening effect [39, 71, 72]. On the other hand, in patients with essential hypertension, it reduced PWV without influencing Aix [73].

#### 4.1.8. Nitrates

Nitrates have also been studied in this regard. As vasodilating drugs, they influence the smooth muscle cells of large arteries, leading to possible arterial destiffening. Their effect was evaluated in hypertensive patients, where isosorbide mononitrate proved to amplify systolic and pulse pressure but substantially reduced Aix. On the other hand, they did not influence PWV, therefore leading to the conclusion that their effect on arterial stiffness was minimal [96].

It seems that arterial stiffness reduction can be obtained with different antihypertensive drugs. The highest effects are exerted by inhibitors of the renin-angiotensin system. Stiffness reduction seems to be correlated with the dose of antihypertensive drug. Long-term treatment also seems to have a greater effect. Mechanisms behind this are the slow extracellular matrix turnover, the long-term constant of arterial remodeling, and the necessity that the target tissue systems influence changes in arterial stiffness more than blood pressure [117].

### 4.2. Statins

Statins (HMG-CoA reductase inhibitors), besides their basic action in reducing low-density lipoprotein (LDL) cholesterol, also have several additional protective/beneficial effects (pleiotropic effects) on the cardiovascular system [79]. There are conflicting reports in the literature on whether statins could improve arterial stiffness directly. Rizos et al. reviewed 9 randomized controlled studies (RCT) with 471 participants. In four of them central aortic PWV was assessed; fluvastatin decreased PWV in two studies, whereas in one study the change was not significant and in another study a significant increase in PWV was observed. In the other five studies peripheral (mainly brachial-ankle) PWV was assessed; fluvastatin decreased PWV in all except one study [80]. Arterial stiffness was improved with atorvastatin (40 mg daily) in patients with ischemic heart failure [81]. Low-dose atorvastatin (10 mg daily) was shown to improve arterial stiffness in a double blind, randomized, placebo-controlled study on hypertensive and hypercholesterolemic patients after 26 weeks of treatment [82]. Similarly, it also prevented an increase in arterial stiffness in patients with chronic kidney disease [83]. Statin treatment significantly improved arterial stiffness through decrease of PWV in normotensive patients with coronary artery disease (CAD), but not in hypertensive patients with CAD [84]. The possible mechanisms behind these observed phenomena are that statins act in an anti-inflammatory and antioxidative manner in the arterial wall, which was shown only in relatively small studies [85–87].

### 4.3. Peroral Antidiabetic Drugs

Treatment with glitazones, peroxisome proliferator-activated receptor gamma (PPAR-*γ*) agonists, was shown not only to improve insulin resistance and glycemic control, but also to decrease arterial stiffness in patients with type 2 diabetes mellitus [64, 100, 101]. PPAR receptors were also proven to be expressed in the vascular tissue and influence vascular homeostasis [118]. In the literature it was shown that pioglitazone decreased arterial stiffness in patients with diabetes mellitus type 2 [64] and in obese glucose tolerant men [65]. For rosiglitazone there are conflicting results; in some studies it decreased PWV, which was associated with anti-inflammatory action [100–102]. In another study, eight-week treatment with rosiglitazone failed to improve arterial stiffness in patients with chronic kidney disease, but the study was small (70 patients, divided equally among treatment and placebo groups) [103]. Treatment with pioglitazone and rosiglitazone was associated with improvement in adiponectin levels [66]. Besides glitazones, metformin was also shown to reduce arterial stiffness in several studies, including women with polycystic ovary syndrome [88, 89].

### 4.4. AGE Cross-Link Breakers

Cross-links between collagen and elastin in the vascular wall are important in providing strength and elasticity to the vessels. Due to nonenzymatic glycation of proteins, especially collagen, AGE are formed, which accumulate and increase collagen cross-linking and consequently progressively increase arterial stiffness. Formation of AGE occurs with aging and is accelerated in diabetes mellitus and hypertension [119]. Newer therapeutics are directed at the cross-linking of collagen, a process previously thought to be irreversible. They can either block the formation of AGE (aminoguanidine) or nonenzymatically break AGE cross-links (alagebrium chloride) and could potentially decrease arterial stiffness [120]. Aminoguanidine was tested mainly in animal studies, where it decreased arterial stiffness parameters [67–69], and only in one human study [70]. Alagebrium chloride (ALT-711) was shown to decrease arterial stiffness in several animal and human studies [16, 90, 91], but not in all [92]. Drugs that block the receptor for AGE (RAGE), or serve as a sham RAGE, are under development [120].

### 4.5. Anti-Inflammatory Drugs

Inflammation states, such as inflammatory bowel disease [121, 122], rheumatoid arthritis, and low-grade systemic inflammation with increased C-reactive protein [123], are associated with the arterial stiffening process. Several anti-inflammatory drugs tested were shown to reduce arterial stiffness [124]. Antibodies against tumor necrosis factor alpha (anti-TNF-*α*) were shown to be effective in improving arterial stiffness in several studies, including patients with chronic inflammatory diseases [104–107]. In contrast, in recent studies they failed to influence arterial stiffness [108–110]. Corticosteroids could also improve arterial stiffness [93]. Conflicting results were also published for acetylsalicylic acid (Aspirin), which could potentially improve arterial stiffness due to its anti-inflammatory effect [111, 112].

### 4.6. Endothelin-A Receptor Antagonists

The serum concentration of endothelin-1 was shown to correlate with aortic elasticity parameters in 152 subjects with essential hypertension [125]. Therefore, selective endothelin-A receptor antagonists could improve arterial stiffness. The beneficial effect of selective endothelin-A receptor antagonists (sitaxsentan, BQ-123) in improving arterial stiffness was shown in studies including patients with chronic kidney disease [94, 95]. Obviously large clinical studies are needed to definitely prove this effect.

### 4.7. Vasopeptidase Inhibitors

Vasopeptidase inhibitors are a new class of drugs that act on two key enzymes in the metabolism of vasoactive peptides: they inhibit ACE to reduce vasoconstriction and inhibit endopeptidase, which is involved in the degradation of several natriuretic peptides, to enhance vasodilation [126, 127]. In a study performed on 167 patients the vasopeptidase inhibitor omapatrilat reduced Aix but failed to influence arterial stiffness [115]. Vascular stiffness was also unaltered by omapatrilat in a study on stroke-prone spontaneously hypertensive rats [116]. Therefore, further studies are needed to investigate the influence of vasopeptidase inhibitors on arterial stiffness.

## 5. Emerging Pharmacological Approaches

New, innovative therapeutic pharmacological options are emerging and show some promise [128]. Short term, low-dose treatment with a statin and sartan separately, but particularly their combination, was shown to improve endothelial function and arterial stiffness parameters in apparently healthy participants, as well as in patients with diabetes mellitus type 1 [129–132]. This approach is oriented directly to arterial function improvement and represents a simple preventive approach against arterial aging.

## 6. Conclusion

Arterial stiffness progressively increases with age and was found to be a risk factor of cardiovascular disease. Therefore, besides identifying solely classical cardiovascular risk factors, it appears to be more and more important to assess arterial stiffness, which could be easily measured noninvasively and expressed as PWV. This modified or advanced approach enables better cardiovascular risk stratification. We believe that such an approach should be introduced into the treatment and prevention of cardiovascular disease. At the present there are several pharmacological agents which could influence arterial stiffness. Consequently, the present review focused on a survey of different pharmacological therapeutic options for decreasing arterial stiffness. However, new and more effective treatment is highly desirable. Furthermore, future studies should address the influence of decrease in arterial stiffness on major adverse cardiovascular and cerebrovascular events (MACCE).

## Figures and Tables

**Table 1:** Influence of particular drugs or drug groups on arterial stiffness. Uniform effect—the drug or drug group definitely improves arterial stiffness; prevailing effect—the drug or drug group improves arterial stiffness in the majority of studies; conflicting effect—the drug or drug group effect is homogeneously distributed between improving arterial stiffness or not; neutral effect—the drug or drug group does not influence arterial stiffness.

Effect on arterial stiffness reduction/improvement	Drug group	Drug class/drug	References
Uniform effect	Antihypertensive	Angiotensin converting enzyme inhibitors	[25–40]
		Angiotensin receptor blockers	[38, 40–54]
		Calcium channel blockers	[32, 34, 37, 46, 49, 55–60]
		Aldosterone antagonists-spironolactone	[61–63]
	Peroral antidiabetic drugs	Glitazones-pioglitazone	[64–66]
	AGE cross-links breakers	Aminoguanidine	[67–70]

Prevailing effect	Antihypertensive	Direct renin inhibitors	[39, 71–73]
		Beta-blockers	[27, 39, 53, 54, 74–78]
	Lipid lowering drugs	Statins	[79–87]
	Peroral antidiabetic drugs	Metformin	[88, 89]
	AGE cross-links breakers	Alagebrium chloride	[16, 90–92]
	Anti-inflammatory drugs	Corticosteroids	[93]
	Endothelin-A receptor antagonists	Sitaxsentan, BQ-123	[94, 95]

Conflicting effect	Antihypertensive	Nitrates	[96]
		Aldosterone antagonists-eplerenone	[97–99]
	Peroral antidiabetic drugs	Glitazones-rosiglitazone	[100–103]
	Anti-inflammatory drugs	Antibodies against tumor necrosis factor alpha (anti-TNF-*α*)	[104–110]
		Acetylsalicylic acid	[111, 112]

Neutral effect	Antihypertensive	Diuretics	[34, 37, 46, 55, 59, 61, 113, 114]
	Vasopeptidase inhibitors	Omapatrilat	[115, 116]
